# Novel lipidomes profile and clinical phenotype identified in pneumoconiosis patients

**DOI:** 10.1186/s41043-023-00400-7

**Published:** 2023-06-15

**Authors:** Liyong Shi, Xiaofang Dai, Furong Yan, Yujun Lin, Lianshun Lin, Yongquan Zhang, Yiming Zeng, Xiaoyang Chen

**Affiliations:** Department of Pulmonary and Critical Care Medicine, Respiratory Medicine Center of Fujian Province, Second Affiliated Hospital of Fujian Medical University, Quanzhou, Fujian Province China

**Keywords:** Pneumoconiosis, Lipidomics, Phenomes, Trans-omics

## Abstract

**Background:**

Pneumoconiosis is a group of occupational lung diseases caused by the inhalation of mineral dust in the lungs, leading to lung dysfunction. Patients with pneumoconiosis are usually accompanied by weight loss, which suggests a lipid metabolism disorder. Recent progress in lipidomics uncovered detailed lipid profiles that play important roles in respiratory diseases, such as asthma, lung cancer and lung injury. The purpose of this study was to shed light on the different expression of lipidome between pneumoconiosis and healthy, hoping to bring new ideas for the diagnosis and treatment of pneumoconiosis.

**Methodology:**

This non-matching case–control study was performed among 96 subjects (48 outpatients with male pneumoconiosis and 48 healthy volunteers), data of clinical phenotypes were recorded, and plasma biochemistry (lipidomic profiles) was tested for both pneumoconiosis patients and healthy controls. A total of 426 species in 11 lipid classes were analyzed by high-performance liquid chromatography coupled with triple quadrupole tandem mass spectrometry (HPLC-QqQ-MS) for the cases and controls. We also analyzed the correlation of lipid profiles with clinical phenomes from pneumoconiosis patients by expression quantitative trait locus (eQTL) model to evaluate trans-nodules between lipidomic profiles and clinical phenomes. All visually re-checked data were analyzed using appropriate statistical tools (*t*-test or one-way ANOVA test) on SPSS.

**Results:**

Compared with healthy people, 26 significantly increased (> 1.5-fold) and 30 decreased lipid elements (< 2/threefold) in patients with pneumoconiosis were identified (*P* values all < 0.05). The majority of those elevated lipid elements were phosphatidylethanolamines (PEs), and the minority were free fatty acids (FFAs), while phosphatidylcholines (PCs) and lysophosphatidylcholines (lysoPCs) declined in pneumoconiosis. Clinical trans-omics analyses demonstrated that phenomes in pneumoconiosis connections with multiple lipids, which showed that pH, lung function, mediastinal lymph node calcification, and complication were highly correlated with lipid elements. Furthermore, up-regulated PE was corresponded to pH, smoking history and mediastinal lymph node calcification. PC was corresponded to dust exposure history, BMI and mediastinal lymph node calcification.

**Conclusion:**

We found altered lipid panels between male pneumoconiosis patients and healthy people by qualitatively and quantitatively measured plasma lipidomic profiles. The trans-omic analysis between clinical phenomes and lipidomes might have the potential to uncover the heterogeneity of lipid metabolism of pneumoconiosis patients and to screen out clinically significant phenome-based lipid panels.

**Supplementary Information:**

The online version contains supplementary material available at 10.1186/s41043-023-00400-7.

## Introduction

Pneumoconiosis refers to a series of pulmonary diseases caused by the inhalation of mineral dust, usually found in certain occupations [1]. As it is widespread globally, it poses a life-threatening risk to public health. There were more than 97 5000 cases of occupational illness occurring in 2018, and 90% of reported occupational diseases were pneumoconiosis [2]. Currently, pneumoconiosis diagnosis mainly relies on reliable dust exposure history and chest radiography [3]. However, both methods have low sensitivity and specificity. In China, almost mining industry employees are predominantly men. Therefore, we selected male patients with pneumoconiosis for research.

Pneumoconiosis patients are usually in poor nutritional status and wasting state, especially those with pneumoconiosis-related complications (e.g., tuberculosis, respiratory infection, and pneumothorax) [4, 5]. Previous studies suggest that pneumoconiosis patients have dyslipidemia characterized by decreased levels of serum cholesterol (CHO), triglyceride (TAG), high-density lipoprotein (HDL) cholesterol, and low-density lipoprotein (LDL) cholesterol [6, 7]. Weight loss and poor nutritional status may be related to abnormal lipid metabolism. However, lipidomic profiles between health and pneumoconiosis had never been defined before, and the potential role of lipids in pneumoconiosis pathogenesis remains unclear.

Lipids are the fundamental components of biological membranes as well as the metabolites of organisms, which play critical roles in cellular energy storage, structure, and signaling [8, 9]. Lipidomics has been accepted as a lipid-related research tool in numerous human lifestyle-related diseases, such as atherosclerosis [10], diabetes [11], Alzheimer’s disease [12], lung transplantation [13], and cancer [14]. Progress in lipidomic technology has uncovered detailed lipid profiles and revealed that lipids were involved in the pathophysiology of human respiratory diseases. Sterols, such as cholesterol, steroid hormones, and bile acids, had been shown to play an important role in lung diseases, including acute respiratory distress syndrome (ARDS) [15], asthma [16–18], COPD [19], and so on. Therefore, lipids play important roles in lung diseases. With the rapid development of biotechnology in analytical chemistry, lipidomics has been used as a tool to define the lipid profiling of lung tissue and plasma, and the relationship between lipid characterization and lung function [20, 21]. To date, little is known about the role and impact of the lipidome in pneumoconiosis. This method of using untargeted lipidomics in HPLC-QqQ-MS provides hope for solving the clinical problems of pneumoconiosis.

Clinical trans-omics is defined as a new subject to integrate clinical phenomes with multi-omics in multiple dimensions [22]. With the rapid development of omics understanding and biotechnology, there was a lack of research in linking the lipidome data with clinical phenomes. Like other omics analyses, most genomic data were not tied with clinical information, which showed limited value for clinical precision medicine [23]. In order to integrate lipid profiles with clinical phenomes in diseases, clinical lipidomics is a novel extension of lipidomics to study lipid profiles, pathways, and networks by characterizing and quantifying the total lipidomes in patients and linking the lipidomics components to clinical phenomics [24]. Zhu et al. [20] found that chest distress corresponded with PS and PG in non-small cell lung cancer (NSCLC), whereas PG, lysoPS, and PS in SCLC. It also indicates that lysoPS were involved in the appearance of sputum, dysphagia, and hemoptysis in patients with adenocarcinoma. Telenga et al. [25] indicate CERs and SMs were inversely correlated with forced expiratory volume in the first second (FEV_1_), FEV_1_/forced vital capacity (FVC), and residual volume/diffusion capacity in smokers with and without COPD, while one CER was associated with diffusion capacity for carbon monoxide. Therefore, the integration of lipidomics with clinical phenomes will provide a novel insight into understanding the molecular mechanisms of diseases [26, 27].

We hypothesized that exploration of abnormal lipid metabolism in pneumoconiosis may contribute to understanding the pathogenesis and prevention of pneumoconiosis. This research was to explore and analyze the differences in lipidomic profiles between patients with pneumoconiosis and healthy people using untargeted lipidomics in HPLC-QqQ-MS. In addition, we conducted the clinical trans-omics to reveal the correlation between clinical phenome and specific groups of lipid elements.

## Methods and materials

### Study design and setting

The study, designed as a retrospective hospital-based non-matching case–control approach including 48 male pneumoconiosis patients and 48 healthy controls, was conducted in Fujian Region of China, between September 2019 to September 2020. Subjects were recruited from the 2nd Affiliated Hospital of Fujian Medical University. The study protocol was approved by the Ethical Evaluation Committee of the 2nd Affiliated Hospital of Fujian Medical University in China (ethical code B2020-293). Written informed consent for clinical data collection and general information questionnaires, including demographic information, history of any diseases, and current treatments before starting the study was obtained from all participants, who were assured of the confidentiality of the information provided and their right to withdraw at any time without prejudice.

### Sample size

The sample size was determined as a pilot using a 90 percent power and a 0.5 effect size. The sample size was determined to be 27 people. With a 10% dropout rate, the final sample size was calculated to be at least 60 participants (30 in each group) [28].

### Participants

We recruited 96 male adults, 48 male patients with pneumoconiosis (randomly selected from Pulmonary and Critical Care Medicine of 2^nd^ Affiliated Hospital of Fujian Medical University) were diagnosed by dust exposure history and chest imaging or pathology. Other 48 male healthy volunteers were recruited prospective randomly from the medical examination center of the same hospital who met the following criteria: having a BMI ranging from ≥ 18.5 to 27.5, and no history of respiratory or other diseases likely to interfere with the study.

### Inclusion and exclusion criteria

Inclusion criteria included participants aged 30 to 80 years, providing written, informed consent for participation in the study. The current criteria of pneumoconiosis were compiled by the International Labour Organization (ILO) [29]. The process of diagnosis was summarized as material submission, medical examination, and diagnosis conditions including confirmation that harmful dust exposure, consistent chest radiographic abnormalities with pneumoconiosis and the absence of other illnesses with similar clinical features (e.g., pulmonary fungal infection, tuberculosis). Exclusion criteria included subjects with other lipid metabolism disorders such as hyperthyroidism, diabetes mellitus, liver or kidney diseases, neoplastic diseases and communicable diseases such as active tuberculosis. Other exclusion criteria also included an intention to lose weight.

### Data collection and clinical phenotypes

Both the pneumoconiosis patients and the healthy controls were interviewed using a pre-tested semi-structured questionnaire, and then the I.V. blood samples (2 ml per person) were collected in the hospital. Fasting blood was drawn from healthy controls and pneumoconiosis patients on the day of visiting our hospital to harvest plasma. All the medical records, including symptoms, signs, laboratory tests, images, and pathologic information were collected. A total of 15 clinical phenomes were collected for the pneumoconiosis group, it mainly consists of the following parts:

1. Medical history including the history of dust exposure, smoking, and pneumothorax was recorded at the first visit. (1) A clear history of exposure to harmful dusts and exposure time was recorded. (2) Previous and current history of pneumothorax were evaluated by past history and chest imaging. (3) Never-smokers were subjects who had never smoked for 1 year or more, and had a total cigarette exposure of less than 0.5 pack-years while the others were smokers.
2. Anthropometric measurements: For body weights were measured in light clothing, without shoes to the nearest 0.1 kg (kg) using a digital balance. Heights were measured to the nearest 0.1 cm (cm) using the SECA balance with height attachment. BMI cut-offs for Asians proposed by the International Obesity Task Force (IOTF-2000) were used for identifying overweight and obesity which was; < 18.49 kg/m^2^ for underweight, 18.5 to 22.9 kg/m^2^ for normal weight, 23 to 27.49 kg/m^2^ for overweight, and > 27.5 kg/m^2^ which indicates obese [30].
3. Laboratory measurements and Supplementary Examination: (1) The fasting serum lipid profile (cholesterol, triglyceride, and HDL) was determined by the enzymatic colorimetric method. (2) The assessment of arterial blood gas (ABG) analysis (PAPIDPoint 500, SIEMENS, Germany) was conducted by the laboratory department including the potential of hydrogen (pH), and arterial carbon dioxide partial pressure (PCO_2_), and arterial oxygen partial pressure (PO_2_). (3) Lung function analysis (PowerCube Body, GANSHORN Medizin Electronic, Germany) was applied for measuring FEV_1_, FVC, forced expiratory one-second rate (FEV1%, FEV_1_/FVC), total lung capacity (TLC).
4. Others: (1) Mediastinal lymph node calcification was assessed by chest imaging. (2) The diagnostic criteria of respiratory failure were PaO_2_ less than 60 mmHg under resting air inhalation, with or without PaO_2_ ≥ 50 mmHg. (3) Normal spirometry was defined as post-bronchodilator FEV_1_ ≥ 80%predicted, post-bronchodilator FEV_1_/FVC > lower limit of normal, and reversibility to 400 µg salbutamol, 10% of the predicted FEV_1_). Less than 80% of TLC was defined as restrictive ventilation dysfunction. (4) mMRC questionnaire for assessing the severity of dyspnea by symptoms [31]. (5) Complications, including COPD, Chronic pulmonary heart disease, pneumothorax, tuberculosis, and respiratory infection.

### Lipid extraction for mass spectrometry analysis

#### Chemical agents

The internal standard mix was subscribed from the Internal Standards Kit for Lipidyzer™ Platform (SCIEX, MA, USA). The acetonitrile, water, isopropanol, ammonium acetate and ammonium hydroxide were HPLC grade and obtained from Sigma-Aldrich (St. Louis, MO, USA) [32].

#### Lipid extraction for mass spectrometry analysis

The plasma samples (20 μL each) were precipitated by the addition of isopropanol (400 μL each) which was precooled to 4 °C and then mixed with lipid internal standard mix. Samples were vortex mixed for 1 min. After 10 min of incubation at room temperature, samples were stored overnight at − 20 °C to improve protein precipitation. The next day, the sample was centrifuged at 14 000 g for 15 min. The supernatant was collected (200 μL) in a sample injection bottle and stored at − 80 °C awaiting Mass Spectra (MS) analysis [32].

#### Identification of lipidomic profiles

The mass spectrometry analysis was performed on a Lipidyzer™ Platform, including the Sciex QTRAP® (SCIEX, Darmstadt, Germany) 4500 system. Multiple reaction monitoring (MRM) was used to target and quantitate several hundreds of lipids molecular. All samples were first measured in positive and negative polarity with SelexION separation, followed by measurement without SelexION separation. Lipids were identified based on their retention time, and mass spectral information. We provide this information (filename-identification method.xlsx). All data were acquired and processed automatically using the Lipid Manager Workflow software (SCIEX, Darmstadt, Germany).

#### Comprehensive analyses of lipidomic profiles

MultiQuant software (AB SCIEX) was used to process data after lipids were identified by mass spectrometry. Further, MetaboAnalyst software 5.0 (www.metaboanalyst.ca) was utilized for conducting multivariate statistical analysis, cluster analysis, dimensionality reduction, and making heat maps.

### Trans-nodule analyses cross phenome and lipid network layers

Our study made the descriptive clinical phenomes scored by the DESS system [33] in the pneumoconiosis group, converting descriptive information of clinical phenomes into digital information [34], based on the clinical trans-omics principle [24]. The type-specific lipids were identified as more than one and a half times elevated or declined significantly compared with pneumoconiosis subtypes (fold change > 1.5 and *P*-value < 0.05). The expression quantitative trait locus (eQTL) model was utilized to evaluate trans-nodules between lipidomic profiles and clinical phenomes.

### Statistics analysis

Data were presented as mean ± SD. The means of each group were used for calculation and comparison. The research data were subjected to Principal Component Analysis (PCA), Orthogonal signal correction Partial Least Square Discrimination Analysis (OPLS-DA), and Variable Importance in Projection (VIP) with the SIMCA 14.1 software program (Umetrics AB, Umea, Sweden). Statistical significance of differences between two groups was determined by Student’s t-test. Statistical significance was affirmed when *P*-value < 0.05. We ranked 15 clinical phenomes of pneumoconiosis patients. Volcano maps showed significantly elevated or declined lipids in pneumoconiosis patients. A VIP plot was further exploited to sort the lipids according to their importance to differentiate groups. To explore the correlation between lipid elements and clinical phenomes, the lipid-quantitative trait loci model modified from eQTL model was applied.

## Results

### Subject characteristics

Forty-eight male patients with pneumoconiosis were enrolled in the present study, and 48 controls with no history of dust occupational exposure. As shown in Table 1, pneumoconiosis patients had significantly lower levels of serum CHO, TG, HDL, and LDL, and lower BMI than healthy groups than control groups (*P* < 0.05). However, no difference existed in age and smoking status. Of pneumoconiosis patients, there were 50% with complication(s), 56.25% with mediastinal lymph node calcification on chest CT, 60.42% were smokers, 68.75% had a dust exposure time of more than 15 years, 79.17% with abnormal lung function, 37.50% with respiratory failure, 54.16% with BMI ≤ 18.4. The clinical phenomes of pneumoconiosis are BMI, history of dust exposure, smoking and pneumothorax, lung function, FEV_1_, FEV_1_%, pH, PCO_2_, PO_2_, respiratory failure, restrictive ventilation dysfunction, mediastinal lymph node calcification, complications, and mMRC scores (Additional file 1: Table S1).

**Table 1:** Demographics and characteristics of patients with pneumoconiosis as compared with healthy control (*P*-values)

Characteristics	Pneumoconiosis (n = 48)	Healthy (n = 48)	t/χ2-value	*P*-value
Age, year	57.31 ± 10.04	53.67 ± 9.98	1.784	0.078
BMI, kg/m2	19.20 ± 2.45	22.91 ± 2.87	− 6.799	< 0.001
Smoking status				
Non-smoker	17 (35.4%)	22 (45.8%)	1.080 (χ2)	0.299
Smoker	31(64.6%)	26 (54.2%)		
T-Cho, mg/dl	4.63 ± 0.90	5.50 ± 0.80	− 5.050	< 0.001
TG, mg/dl	1.12 ± 0.40	1.67 ± 1.08	− 3.276	0.002
HDL-Cho, mg/dl	1.21 ± 0.33	1.40 ± 0.37	− 2.621	0.010
LDL-Cho, mg/dl	3.27 ± 0.90	3.66 ± 0.60	− 2.518	0.014

### Plasma lipidomic profiles of patients with pneumoconiosis

**Fig. 1:**
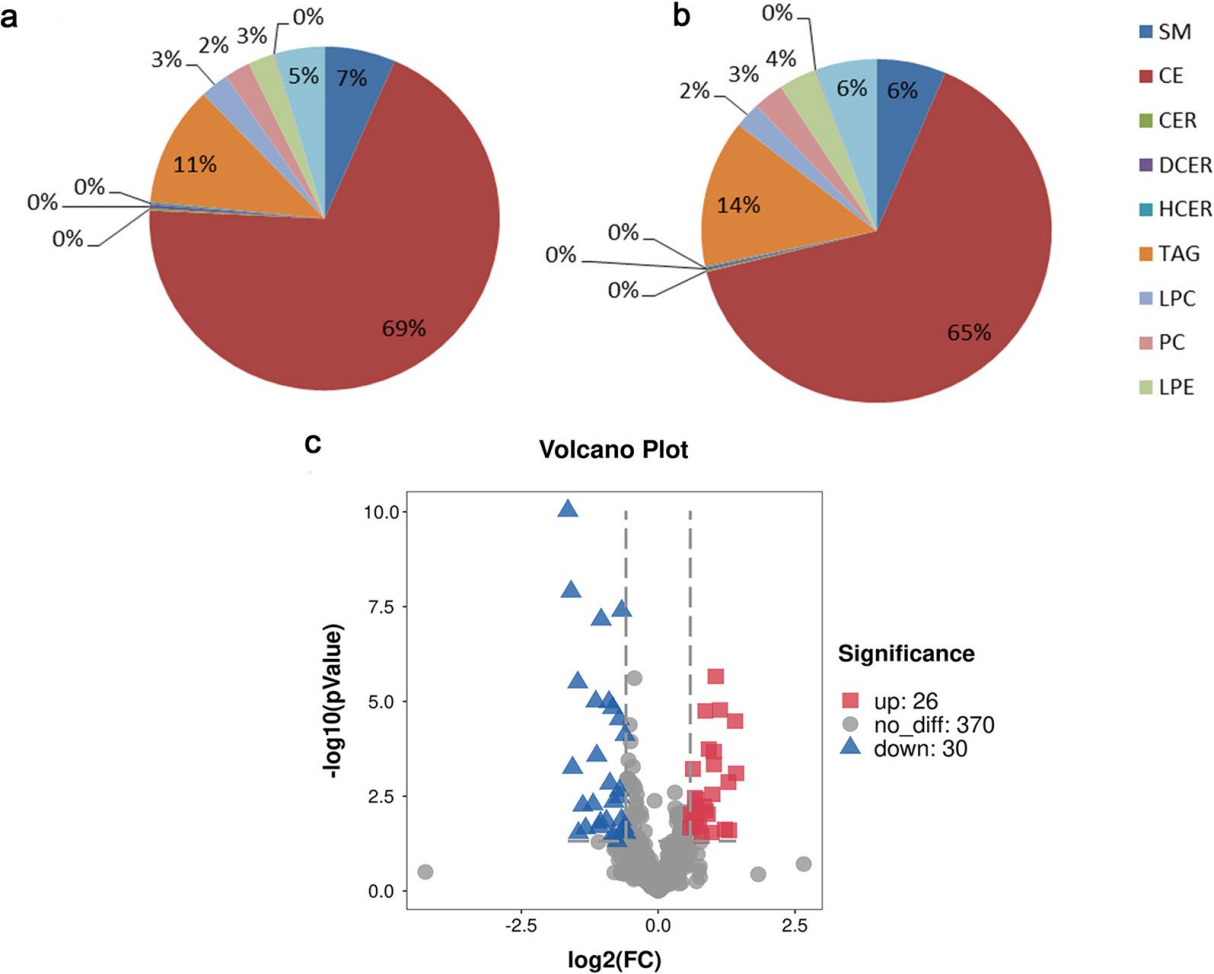
The lipid classification of differential metabolites. The proportion (%) of 11 main lipid elements of healthy controls (**a**) and pneumoconiosis (**b**). Volcanic map (**c**) between healthy controls with pneumoconiosis. The abscissa represents log values of fold changes, where the left side of the first dotted line perpendicular to the abscissa represents < 2/threefold changes (down-regulation) and the right side of the second dotted line represents > 1.5-fold changes (up-regulation). The vertical coordinate represents –log10 (*P*-value). The upper side of the dotted line perpendicular to the ordinate stands for P-value less than 0.05, as compared with healthy controls

A total of 426 lipid elements of plasma were identified qualitatively and quantitatively. The main lipid subclasses mainly including subclasses of phosphatidic acid (PA), phosphatidylcholine (PC), phosphatidylethanolamine (PE), phosphatidylglycerol (PG), phosphatidylinositol (PI), and phosphatidylserine (PS). All the identified lipid species were grouped into 11 lipid classes, mainly including 107 PEs, 65 PCs, 12 SMs, 3 cholesterols (CEs), 3 CERs, 2 diacylceramides (DCERs), 5 hexosylceramides (HCERs), 195 tri-acylglycerols (TAGs), 16 lysophosphatidylcholines (LPCs), 1 lysophosphatidylethanolamine (LPE), 17 FFAs (Fig. 1). For the proportion (%) of those 11 lipid classes, healthy controls and pneumoconiosis patients were shown in Fig. 1a and b. The volcanic map demonstrated the clear patterns of lipid elements significantly increased and declined between healthy controls and pneumoconiosis in Fig. 1c. The lipid elements were identified on the basis of statistical significance. 26 significantly increased and 30 decreased lipid elements in patients with pneumoconiosis were identified (detail list in Table 2). It was noticed that the majority of those elevated lipid elements were PE (69%) and FFA (15%), while PC (23%), TAG (23%), and LPC (17%) declined in pneumoconiosis.

**Table 2:** Lipid elements significantly elevated and declined alone in patients with pneumoconiosis as compared with healthy control (*P*-values)

Elevated > 1.5 fold			Declined < 2/threefold		
Lipids	Fold	*P*-value	Lipids	Fold	*P*-value
PE(16:0/16:1)	2.68	0.00	PC(18:1/20:5)	0.67	0.03
PE(18:1/18:3)	2.65	0.00	LPC(20:0)	0.66	0.00
FFA(20:4)	2.46	0.03	TAG(58:7/FA18:0)	0.65	0.02
FFA(22:4)	2.43	0.00	PC(16:0/20:5)	0.64	0.03
TAG(55:4/FA18:2)	2.33	0.02	LPC(18:0)	0.63	0.00
PE(18:1/18:2)	2.19	0.00	TAG(58:9/FA18:1)	0.63	0.01
PE(18:1/18:1)	2.07	0.00	PC(14:0/22:6)	0.61	0.00
PE(16:0/18:3)	2.03	0.00	LPC(22:6)	0.61	0.00
PE(18:2/18:2)	2.02	0.00	PE(14:0/20:4)	0.61	0.00
FFA(20:5)	1.99	0.00	TAG(56:6/FA22:6)	0.59	0.05
PE(O-18:0/22:4)	1.97	0.03	PC(18:2/20:5)	0.57	0.00
PE(16:0/18:2)	1.90	0.00	TAG(56:7/FA16:0)	0.57	0.04
FFA(20:3)	1.88	0.01	CER(24:1)	0.56	0.00
PE(16:0/22:4)	1.83	0.01	TAG(58:7/FA16:0)	0.55	0.04
PE(16:0/18:1)	1.82	0.00	PC(20:0/22:6)	0.54	0.00
PE(18:2/20:2)	1.79	0.01	LPC(14:0)	0.54	0.00
PE(18:0/22:4)	1.73	0.04	TAG(55:1/FA16:0)	0.52	0.01
PE(18:0/16:1)	1.68	0.02	HCER(24:1)	0.49	0.00
PE(P-18:0/22:4)	1.63	0.00	PC(14:0/20:5)	0.48	0.02
TAG(55:4/FA18:1)	1.63	0.02	TAG(56:8/FA16:0)	0.47	0.02
PC(16:0/16:1)	1.61	0.01	LPC(20:5)	0.46	0.00
PE(18:0/16:0)	1.59	0.00	HCER(20:1)	0.45	0.00
PE(18:0/18:3)	1.56	0.02	PE(P-18:0/20:5)	0.44	0.01
PE(18:1/20:4)	1.55	0.00	PE(P-18:1/20:5)	0.40	0.02
PE(18:1/20:3)	1.51	0.01	PE(O-16:0/20:5)	0.39	0.01
PC(18:0/16:1)	1.50	0.02	PE(P-16:0/20:5)	0.36	0.03
			DCER(22:0)	0.36	0.00
			PC(20:0/20:5)	0.34	0.00
			CER(22:1)	0.33	0.00
			HCER(22:1)	0.32	0.00

**Fig. 2:**
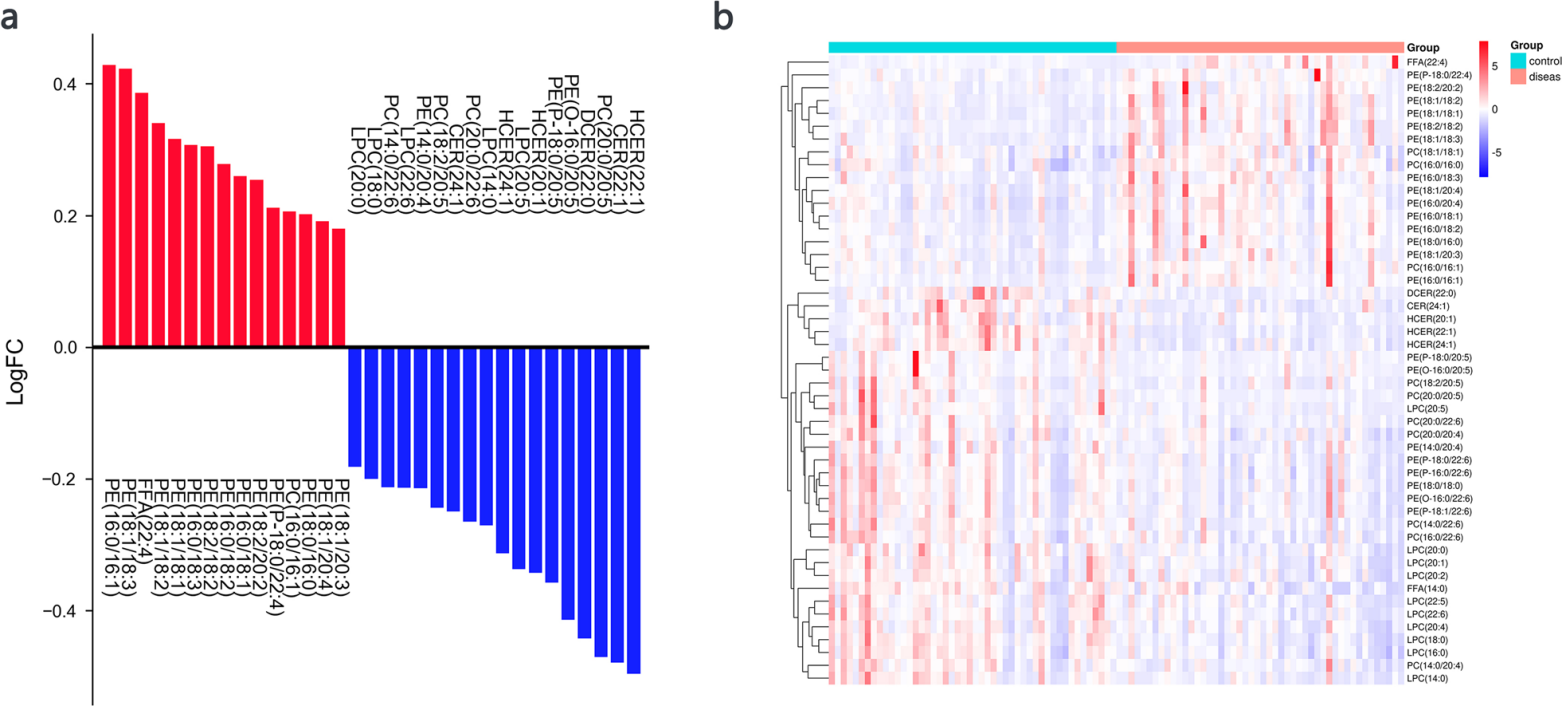
**a** Comparison of lipid metabolites between the pneumoconiosis and control. The bar plot describes the lipid elements that increased and declined in pneumoconiosis patients compared with healthy control among the top 50 lipid elements increased from blue (low) and red (high). **b** Identification of differential lipid metabolites between the pneumoconiosis and control group. The heatmap describes the top 50 lipid elements at the high concentration and the degree of lipid elements increased from blue (low) and red (high)

By orthogonal partial least squares discrimination analysis (OPLS-DA), variable important in project (VIP) scores were used to select important lipids. The top 50 lipid elements were defined based on VIP scores mainly distributed in lipids classes of 22 PEs, 10 PCs, 10 LPCs, 2 CERs, 3 HCERs, 1 DCER, and 2 FFAs. Of the top 50 lipids, there were 15 lipid elements significantly elevated (> 1.5 fold) and 18 declined (< 2/threefold) in pneumoconiosis patients compared with healthy control among the top 50 lipid elements (Details listed in Table 3 and the bar plot in Fig. 2a). There was also a clear distribution of the top 50 lipid elements in the pneumoconiosis group, compared with the healthy control, described in a heatmap (Fig. 2b).

**Table 3:** Lipid elements significantly elevated and declined in pneumoconiosis patients compared with healthy control among the top 50 lipid elements defined on VIP scores (*P*-values)

Elevated > 1.5 fold			Declined < 2/threefold		
Lipids	Fold	P-value	Lipids	Fold	P-value
PE(16:0/16:1)	2.68	0.00	LPC(20:0)	0.66	0.00
PE(18:1/18:3)	2.65	0.00	LPC(18:0)	0.63	0.00
FFA(22:4)	2.43	0.00	PC(14:0/22:6)	0.61	0.00
PE(18:1/18:2)	2.19	0.00	LPC(22:6)	0.61	0.00
PE(18:1/18:1)	2.07	0.00	PE(14:0/20:4)	0.61	0.00
PE(16:0/18:3)	2.03	0.00	PC(18:2/20:5)	0.57	0.00
PE(18:2/18:2)	2.02	0.00	CER(24:1)	0.56	0.00
PE(16:0/18:2)	1.90	0.00	PC(20:0/22:6)	0.54	0.00
PE(16:0/18:1)	1.82	0.01	LPC(14:0)	0.54	0.00
PE(18:2/20:2)	1.79	0.01	HCER(24:1)	0.49	0.00
PE(P-18:0/22:4)	1.63	0.00	LPC(20:5)	0.46	0.00
PE(16:0/16:1)	1.61	0.01	HCER(20:1)	0.45	0.00
PE(18:0/16:0)	1.60	0.00	PE(P-18:0/20:5)	0.44	0.00
PE(18:1/20:4)	1.55	0.00	PE(O-16:0/20:5)	0.39	0.00
PE(18:1/20:3)	1.51	0.01	DCER(22:0)	0.36	0.00
			PC(20:0/20:5)	0.34	0.00
			CER(22:1)	0.33	0.00
			HCER(22:1)	0.32	0.00

### Trans-omic profiles between clinical phenomes and lipidomes

**Fig. 3:**
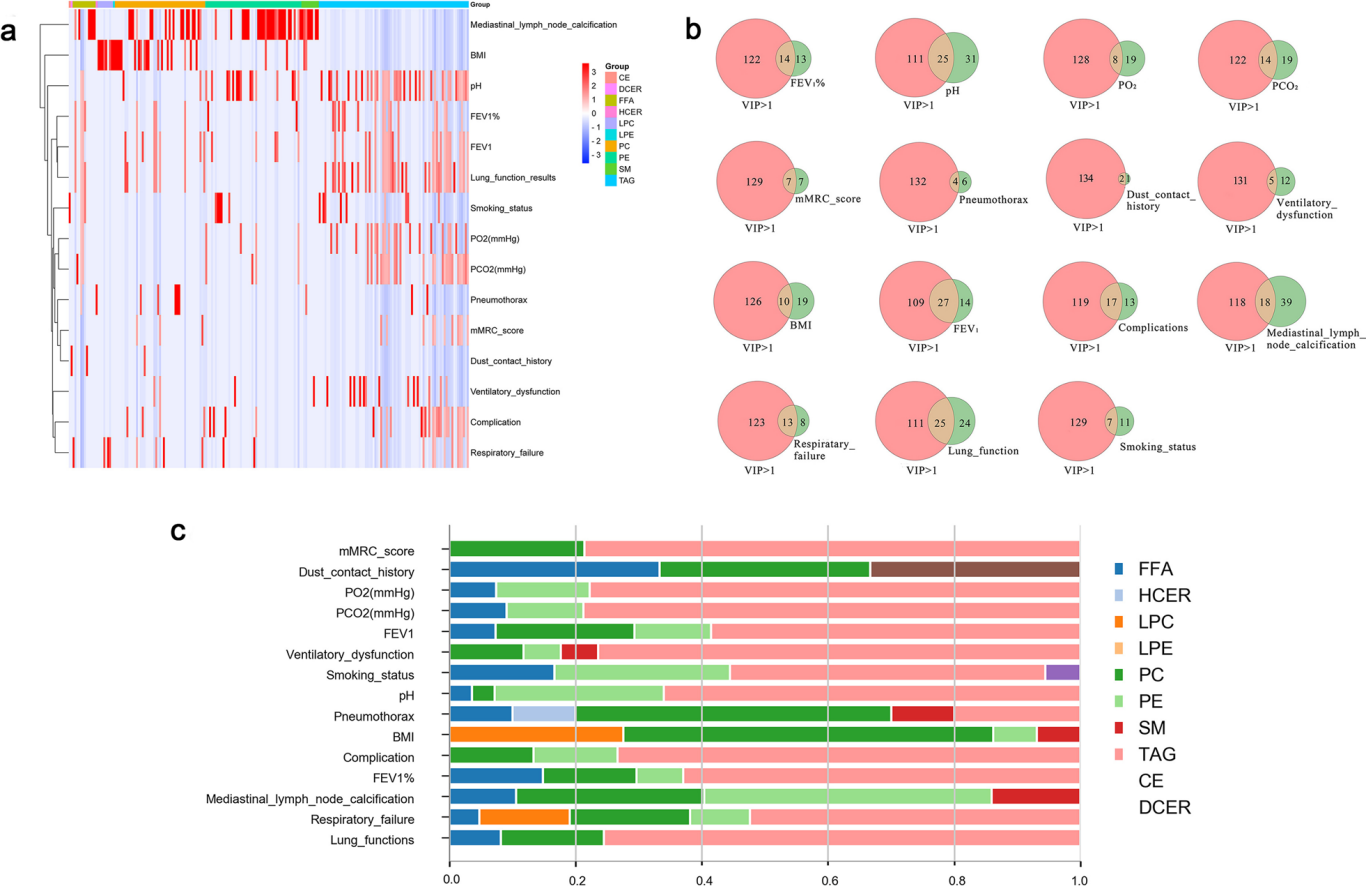
The specific correlation between clinical phenotypes and altered lipid elements of pneumoconiosis patients is illustrated by (**a**), (**b**), and (**c**). **a** Heatmap describes the correlation increased from blue (low) to red (high). **b** Venn's diagrams, the more overlaps, the greater the correlation. **c** Stacked bar plot showing mean relative abundance of lipids in clinical phenomes. The longer column indicates the stronger correlation

The present study initially found the differences in lipidomic profiles among pneumoconiosis and healthy, and also indicates clinical phenome-associated lipids and lipid element-associated clinical phenomes using clinical trans-omics. Circulating levels of lipid elements were integrated with 15 clinical phenomes from patients with pneumoconiosis. Combined lipidomes (VIP > 1) with clinical phenomes were measured by simulating the expression quantitative trait locus (eQTL) model. With the aim to identify disease phenome-specific lipid elements, circulating levels of lipid elements were integrated with clinical phenomes in patients with pneumoconiosis patients. Varied lipid elements and the specific correlated clinical phenotype were displayed in Fig. 3a and b, which showed FEV_1_, complication(s), mediastinal lymph node calcification, and pH were highly correlated with lipid elements (VIP > 1). Furthermore, up-regulated PE was correlated to pH, smoking history, and mediastinal lymph node calcification. PC was correlated to dust exposure history, BMI, and mediastinal lymph node calcification (Fig. 3c).

## Discussion

Lipid plays an important role in lung pathology and the physiology of pneumoconiosis. In the present research, lipidomics was used to explore to investigate the differential lipidomics metabolites in pneumoconiosis. Recent researches indicate these lipids were associated with various respiratory diseases.

PCs and PEs are the most abundant phospholipids in cell membranes and play essential structural and signaling roles. PCs play significant roles in lung surfactant phospholipid metabolism and are the major phospholipid comprising around 80% of surfactant lipids. Alterations in their composition can cause reduced elasticity, leading to an overall decrease in lung compliance [35, 36]. Shabarinath et al. [37] found that phospholipids including PC have been found to increase in pulmonary fibrosis, and the up-regulated PC might be partially associated with greater epithelial injury in the progressive group relative to stable. Meanwhile, Lv et al. [38] demonstrated that levels of PC and lysoPC were increased while PE and lysoPE were decreased in patients with lung cancer. Gao et al. [21] also found that there was an increase of PC and lysoPC in acute exacerbation of chronic pulmonary (AECOPD), acute pulmonary embolism (APE), and severe acute pneumonia (SAP). In the above diseases, the changes trend in PE, lysoPE, PC, and lysoPC are consistent basically. However, the trend of PE, PC, and lysoPC in the pneumoconiosis group in our research was opposite to the results of the above pulmonary diseases. The up-regulated PE and down-regulated PC in our study might be a potential criterion to distinguish pneumoconiosis from healthy and even other lung diseases. Although the reason and mechanism of changed lipidomics in pneumoconiosis are not clear, it may be of great significance to find the pathogenesis of pneumoconiosis and the underlying mechanism needs to be further studied.

In the present study, only one lipid metabolite in the subclass of FFAs, FFA (22:4), was found that significantly higher in pneumoconiosis than in healthy control. Boden et al. [39] reported that elevated plasma FFA levels resulted in increased activation of the classic proinflammatory IKK/IκB/NF-κB pathway and increased expression of inflammatory cytokines (tumor necrosis fator-α, interleukin-1β and interleukin-6). Miyauchi et al. [40] indicated that activation of free FFA4, which is a member of the G protein-coupled receptor family (previously known as GPR120), that responded to FFA, resulted in a decrease in lung resistance. At present, researches on FFA and FFA receptors are mostly focused on metabolic disorders such as diabetes and obesity, whereas little attention is paid to respiratory diseases. A recent study found that FFA4 was highly expressed in lung epithelial cells [41], and that FFA acting via FFA4 receptors might have some beneficial effects on airway epithelial repair after naphthalene-induced airway injury [42]. Therefore, FFA (22:4) may also be a potential target for understanding pathogenesis and treating respiratory diseases including pneumoconiosis.

Further analysis through the eQTL model and integrated lipidomic and phenomic profiles, clinical phenotypes were discovered to be associated with a variety of altered lipid elements. Lipidomic profiles had strong correlations with FEV_1_, complication(s), mediastinal lymph node calcification, and pH in pneumoconiosis. Additionally, PE was correlated to smoking history, mediastinal lymph node calcification, and pH, and PC was correlated with BMI, dust exposure history, and mediastinal lymph node calcification separately.

As to PCs, there are few studies on the relationship between lung function and lipids. Only one study revealed that SiO_2_ exposure could result in decreased secretion of 1, 2-Dipalmitoyl-sn-glycerol-3-phosphocholine (DPPC), which was known as a lipid marker of alveolar type 2 epithelial cells, affecting the normal function of alveoli, and lung function potentially [43]. This prompts that the lipid metabolism disorder may be related to the decline of lung function, particularly FEV_1_. The present study indicated that PC was also correlated with BMI. Some studies confirmed that altered lipid metabolism (PCs and lysoPCs) were associated with lean body mass [44, 45]. These studies were consistent with our experimental results.

PE was found to correlate with not only lung function, but also smoking history in the study. In a study of COPD patients, PEs were found to be higher in smokers, and altered PEs with a high fold change also showed high correlations with lower FEV_1_ and lung function [25]. Results in our study were consistent with the previous study, indicating that PEs were highly expressed in smoking patients. The mechanism and the diagnostic and therapeutic value of elevated PEs were still not clear now. However, there was no report on the association between pneumoconiosis and elevated PEs and smoking history, and the mechanism and diagnostic and therapeutic value of elevated PE was still not clear now, which may be worthy of further study. Recently Wu et al. [46] found that phospholipid remodeling was critical forstem cell pluripotency by facilitating mesenchymal-to-epithelial transition and also showed that PE binding to Pebp1 enhanced the interaction of Pebp1 with IKKα/β and reduced the phosphorylation of IKKα/β and NF-κB signaling. Collectively, studies reveal and highlight the importance of phospholipids in pneumoconiosis, and may provide hope for solving clinical problems and opportunities for in-depth study.

Interestingly, lipids especially PE and PC were both correlated with mediastinal lymph node calcification. Calcification of mediastinal and hilar lymph nodes was commonly found in pneumoconiosis. However, no research into PCs and PEs has been reported for pneumoconiosis disease. We assume that it might be because of macrophage accumulation and altered lipid metabolism in pneumoconiosis present with similar dystrophic calcification. One of the pathophysiological characteristics of pneumoconiosis was macrophage accumulation. Bargagli et al. [47] revealed that autophagy-lysosomal system dysfunction in alveolar macrophages (AMs) play a crucial role in the silica-induced inflammatory and fibrotic process. Another study found that macrophages are associated with hypoxia, glycolytic metabolism, and the production of inflammatory cytokines and revealed that macrophages were fundamental for the repair of organs that were injured due to ischemia [48]. In response to hypoxia, acute-phase macrophages activate hypoxia-inducible transcription factors that adjust cellular metabolism via hglycolysis, the tricarboxylic acid cycle, oxidative phosphorylation, lipids, amino acids pathways leading to metabolic acidosis and decreased pH [49]. The interaction between lipid and protein molecules may be the main reason for the phagocytosis of macrophages. The phagocytosis of phospholipids and CERs can be realized through the modification of the physical properties of the macrophage membrane mediated by phospholipids and the arrangement and domain interaction of lipids [50]. Therefore, the role and mechanism of PC and PE for the calcification of mediastinal lymph nodes in pneumoconiosis need to be further investigated.

There were also limitations in the present study. First, the sample size was small. Second, therapeutic factors were not explored, and most correlating groups of patients should be studied in the future to explore the medical influence on lipidomes. Third, it is not clear whether the correlation is positive or negative by the eQTL model for trans-omics.

In conclusion, we found altered lipid panels between pneumoconiosis patients and healthy people by qualitatively and quantitatively measuring plasma lipidomic profiles. Levels of PE and FFA elements meaningfully improved while PC and lysoPC were reduced in patients with pneumoconiosis. Clinical trans-omics analyses demonstrated that some phenomes have a strong correlation with lipids, although those needed to be further confirmed by bigger studies including a large population of patients in multicenter. Consequently, our data suggested that trans-omic profiles between clinical phenomes and lipidomes might have the potential value to reveal the heterogeneity of lipid metabolism among pneumoconiosis patients and to identify meaningful phenome-based lipid panels as biomarkers.

## Supplementary Information

**Additional file 1. Table S1**. Clinical phenomes scored and collected in pneumoconiosis patients, including BMI, medical history, Laboratory measurements and Supplementary Examinations and others.

## Data Availability

The datasets analyzed during the study are available from the corresponding author on reasonable request and after approval of the concerned authorities in the organization.
