# Supplementary material for: Novel lipidomes profile and clinical phenotype identified in pneumoconiosis patients
Source: J Health Popul Nutr. 2023 Jun 15;42:55. doi: 10.1186/s41043-023-00400-7 (PMC10268378; doi:10.1186/s41043-023-00400-7)
Supplement: Supplementary file 1 — Additional file 1. Table S1. Clinical phenomes scored and collected in pneumoconiosis patients, including BMI, medical history, Laboratory measurements and Supplementary Examinations and others. [file 41043_2023_400_MOESM1_ESM.docx]

Supplement Table 1. Clinical phenomes scored and collected in pneumoconiosis patients, including BMI, medical history, Laboratory measurements and Supplementary Examinations and others.

| **Clinical phenomes** | **DESS Scores** | |
| --- | --- | --- |
|  | 0 | 1 |
| BMI | ≤18.49 kg/m^2^ | >18.49 kg/m^2^ |
| History | | |
| Dust exposure | No | Yes |
| Smoking (pack×year) | <0.5 | >0.5 |
| Pneumothorax | No | Yes |
| Laboratory measurements and Supplementary Examination | | |
| pH | 7.35~7.45 | <7.35 or >7.45 |
| PO_2_ (mmHg) | ≥90 | <90 |
| PCO_2_ (mmHg) | 35-45 | <35 or >45 |
| Respiratory failure | PO_2_＞60mmHg | PO_2_<60mmHg with PCO_2_ ≥50mmHg |
| Lung function | FEV_1_≥80% predicted with FEV_1_/FVC >70% | FEV_1_< 80% predicted with FEV_1_/FVC ≤70% |
| FEV1 | ≥80% predicted | <80% predicted |
| FEV1% (FEV1/FVC) | >70% | ≤70% |
| Restrictive ventilatory dysfunction | TLC≥80% | TLC<80% |
| mMRC score | 0 | 1~4 |
| Mediastinal lymph node calcification | No | Yes |
| Complications | No | Yes |
